# Paratracheal air cyst and bronchogenic cyst in patients with esophageal cancer who received thoracoscopic esophagectomy: A case series of three patients

**DOI:** 10.1016/j.ijscr.2021.106243

**Published:** 2021-07-26

**Authors:** Tomoyuki Okumura, Takeshi Miwa, Toru Watanabe, Takahisa Akashi, Kazuhiro Nomoto, Nana Kimura, Naoya Takeda, Tomofumi Uotani, Hayato Baba, Katsuhisa Hirano, Kazuto Shibuya, Isaya Hashimoto, Shozo Hojo, Koshi Matsui, Isaku Yoshioka, Shigeaki Sawada, Kenichi Tazawa, Fuminori Yamagishi, Tsutomu Fujii

**Affiliations:** a Department of Surgery and Science, Faculty of Medicine, Academic Assembly, University of Toyama, 2630 Sugitani, Toyama 930-0194, Japan; b Department of Surgery, Itoigawa General Hospital, 457-1 Takehana, Itoigawa, Niigata 941-8502, Japan; c Department of Diagnostic Pathology, Faculty of Medicine, Academic Assembly, University of Toyama, 2630 Sugitani, Toyama 930-0194, Japan

**Keywords:** Paratracheal air cysts, Bronchogenic cysts, Esophageal cancer, Thoracoscopic esophagectomy

## Abstract

**Introduction and importance:**

Mediastinal cystic lesions, such as paratracheal air cyst (PTAC) and bronchogenic cyst (BC), are rare anomaly usually found incidentally in thoracic imaging. Special attention is needed in the case of thoracic surgery.

**Case presentation:**

All three patients were male, 71, 73, and 76 years old. Preoperative CT showed each had a lobular cystic lesion at the right posterolateral side of trachea in the thoracic outlet 11, 14, and 19 mm in size, respectively, with air density and tracheal communication, leading to a diagnosis of PTACs. An oval cystic lesion, 7 mm in size, was found in one patient at the right lateral side of the upper esophagus with low density and without tracheal communication, leading to a diagnosis of paraesophageal BC. Intraoperative findings of the three PTACs demonstrated a soft bulge from the membranous portion of trachea that was left intact. The BC had an oval elastic structure, mimicking a metastatic lymph node, and was removed with the mediastinal lymph nodes. Histological examination showed ciliated columnar epithelium, confirming a diagnosis of BC.

**Clinical discussion:**

PTACs are associated with increased intraluminal pressure due to chronic lung disease. BCs are congenital anomalies that originate from abnormal budding of the embryonic foregut.

**Conclusion:**

PTACs and BCs need to be considered in preoperative image diagnosis in patients with esophageal cancer. PTACs should be left intact to avoid tracheal injury, while removal of isolated BCs is recommended as a diagnostic and therapeutic measure.

## Highlights

• Paratracheal air cyst (PTAC) and bronchogenic cyst (BC) are rare anomaly.
• Three PTACs and a BC were found in patients who received thoracoscopic esophagectomy for esophageal cancer.
• PTACs were carefully left intact to avoid tracheal injury.
• BC was removed for diagnostic and therapeutic measure.
• PTACs and BCs need to be considered in preoperative image diagnosis in patients with esophageal cancer.

## 1 Introduction

Small mediastinal cystic lesions, such as paratracheal air cyst (PTAC) and bronchogenic cyst (BC), are rare anomaly which present in 1–4% of the population [1]. They are usually asymptomatic and found incidentally in thoracic imaging. However, special attention is needed in the case of thoracic surgery to prevent tracheal injury [2]. We report here small mediastinal cystic lesions in three patients who received thoracoscopic esophagectomy for esophageal cancer.

## 2 Case presentation

**Fig. 1:**
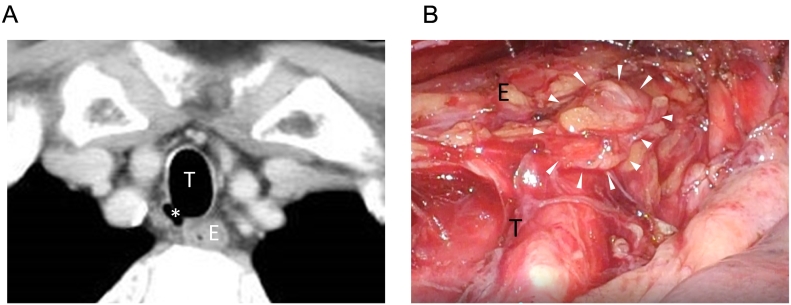
(A) Preoperative CT image of the PTAC in Patient 1 (asterisk). (B) Intraoperative findings of PTAC in Patient 1 (white arrow heads). E: esophagus, T: trachea.

The first patient was a 73-year-old male, who was diagnosed with esophageal squamous cell carcinoma (ESCC) in mid-esophagus, T1bN1M0 StageIIB, using the seventh edition of the Union for International Cancer Control system. He was a heavy smoker with 20 cigarettes per day for 50 years, but without medical history of chronic lung disease, such as chronic obstructive pulmonary disease (COPD) or emphysema. Preoperative computed tomography (CT) detected an oval thin-wall cystic structure between the right posterolateral side of the trachea and the right subclavian artery (Fig. 1A), 11 mm in size, with internal air density and tracheal communication, leading to a preoperative diagnosis of PTAC. During thoracoscopic esophagectomy, PTAC demonstrated a soft bulge from the membranous portion of the trachea that swelled and shrank with ventilation (Fig. 1B). The lymph nodes along the right recurrent laryngeal nerve (RRLN) were removed with careful separation from the PTAC to preserve both the RRLN and PTAC.

**Fig. 2:**
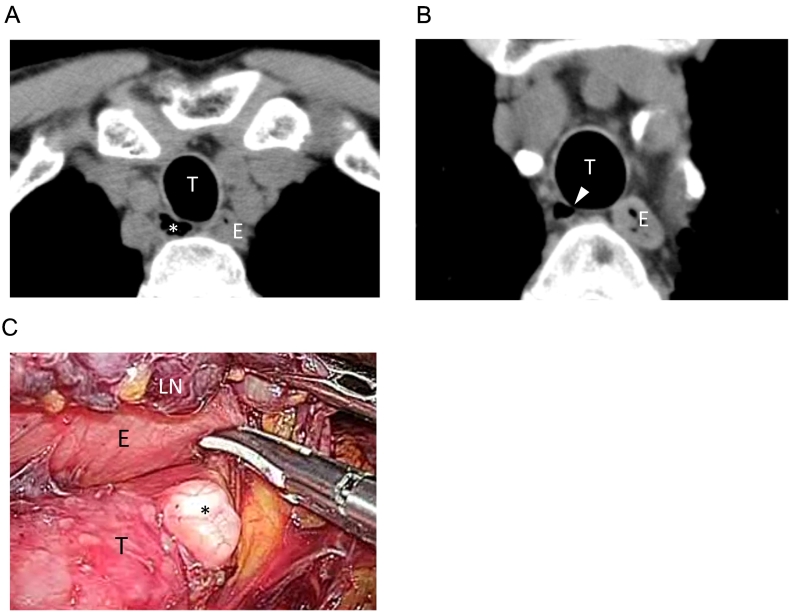
(A) Preoperative CT image of the PTAC in Patient 2 (asterisk). (B) A small connection to the trachea (white arrow head). (C) Intraoperative findings of PTAC in Patient 2 (asterisk). E: esophagus, T: trachea, LN: right para RLN lymph nodes.

The second patient was a 71-year-old male, who was diagnosed with ESCC in lower esophagus, T1bN0M0 Stage I. He was a heavy smoker with 20 cigarettes per day for 45 years, but without medical history of chronic lung disease. CT showed a multilocular cystic structure, 14 mm in size, at the right posterolateral side of trachea at the mid-upper thoracic esophagus (Fig. 2A). The cyst had air density and tracheal communication was seen (Fig. 2B). It was diagnosed as PTAC. During thoracoscopic esophagectomy, the PTAC had an elastic soft wall that attached to the trachea and swelled out and shrank with ventilation. It was left intact with careful separation from the upper esophagus and RRLN lymph nodes (Fig. 2C).

**Fig. 3:**
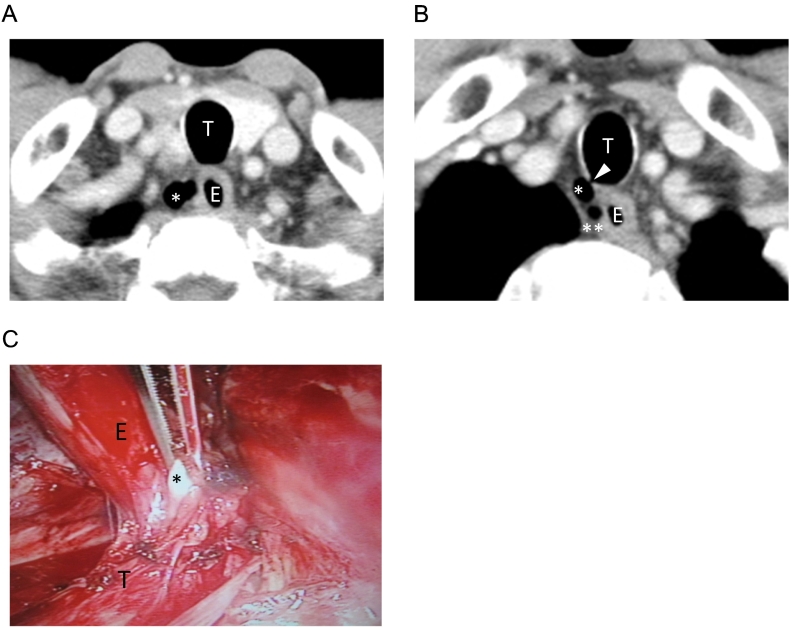
(A) Preoperative CT image of the PTAC in Patient 3 (asterisk). (B) Communication of the PTAC (asterisk) to trachea (white arrow head). An isolated BC was also seen (double asterisks). (C) Intraoperative findings of the BC in Patient 3 (asterisk). E: esophagus, T: trachea.

**Fig. 4:**
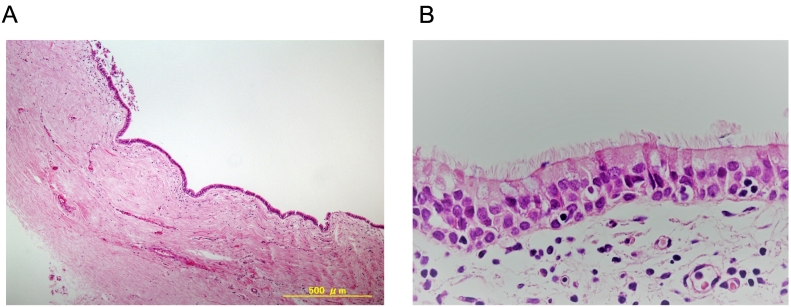
(A) Histological findings of the removed BC in low magnification. (B) Histological findings of the removed BC in high magnification.

The third patient was a 76-year-old male, who was diagnosed with ESCC in mid-lower esophagus, T1bN1M0 StageIIB. He had no smoking history, nor medical history of chronic lung disease. CT showed a multilocular cystic structure in the neck, 19 mm in size, with internal air density (Fig. 3A), which was connected to the right posterolateral side of the trachea (Fig. 3B), leading to a preoperative diagnosis of PTAC. An oval cystic structure, 7 mm in size, was found at the right lateral side of the upper esophagus with low density and without connection to the esophagus or trachea (Fig. 3B), leading to a diagnosis of paraesophageal BC. Intraoperative findings of the PTAC showed a soft bulge from the membranous portion of the trachea. It was left intact during cervical lymph node dissection under direct vision. The BC had a smooth oval elastic structure, mimicking a metastatic lymph node. It was removed along with RRLN lymph nodes during video-assisted thoracoscopic esophagectomy (Fig. 3C). Histological findings demonstrated the presence of ciliated columnar epithelium, confirming the diagnosis of BC (Fig. 4A, B).

All three patients recovered without postoperative complications. No remnant cyst-related complication, no enlargement of the remnant cyst, nor recurrence of the cyst, was observed during 18–115 months follow-up after surgery.

## 3 Clinical discussion

PTACs are air-filled collections adjacent to the trachea and that are sometimes described as tracheal diverticulum [3]. Most PTACs measure approximately 10 mm in size with a direct connection to the trachea and are asymptomatic [3], [4]. They are seen on the right posterior side of the trachea at the junction of the tracheal wall and posterior membrane [3]. Therefore, reports have suggested a relationship between PTACs and prolonged increased intraluminal pressure due to chronic lung diseases such as Chronic Obstructive Pulmonary Disease (COPD) and emphysema [1], [5]. Several reports have also described congenital PTACs with other respiratory tract abnormalities [6]. In the presented cases, location and morphological findings were compatible with typical characteristics of PTACs; however, no medical history of COPD, emphysema, or the other respiratory tract abnormality was found, suggesting unknown pathogenesis of the PTACs.

Asymptomatic small PTACs usually require no treatment [4], [7]. However, as PTACs are commonly found at a lesion where *peri* RRLN lymph node metastasis is frequently seen, preservation of PTACs by careful separation from the esophagus and lymph nodes is necessary to avoid tracheal injury during the esophagectomy [8], [9].

As shown in the above-mentioned reports, many of the PTACs in esophagectomy patients have been reported from Japan. Difference among countries in the incidence of PTACs is unclear. One possible reason is that upper mediastinal lymph node dissection is commonly performed in Japan, where majority of esophageal cancer are squamous cell carcinoma and arise at mid esophagus.

BCs are congenital anomalies that originate from abnormal budding of the embryonic foregut and tracheobronchial tree [10]. Consequently, BCs are commonly found in the mediastinum, including the paratrachea, sub-carina, hilar, and paraesophagus. The content of BC may include mucoid, hemorrhagic secretions, clear fluid, or be air-filled [10]. Although most BCs are asymptomatic and incidentally detected, infection, perforation, and intracystic hemorrhage were reported to be possible complications [11]. Therefore, reports have recommended surgical excision of BCs even in asymptomatic cases to define diagnosis and prevent complications [2], [11]. Minimally invasive surgery under thoracoscopy has been broadly accepted for resection of BCs [11], [12]. In the presented case, the paraesophageal BC was asymptomatic and found incidentally. The BC masqueraded as a metastatic lymph node and was completely removed with para-RRLN lymph nodes during thoracoscopic esophagectomy. Histological observation enabled us to confirm the diagnosis of BC.

Preoperative diagnosis by CT, intraoperative identification, and careful removal of the BCs under consideration of possible communication with the trachea is recommended. However, in the patients presenting with tracheal injury, primary closure or repair using the pericardial or intercostal muscle flap should be considered [13].

There have been reports on PTACs or BCs each, however, to our knowledge, this is the first report that describes PTAC and BC at the same time, suggesting the importance of differential diagnosis and different therapeutic strategies in patients who underwent esophagectomy.

This work was reported in line with the SCARE 2020 criteria [14].

## 4 Conclusion

Small mediastinal cystic lesions, such as PTACs and BCs, need to be considered in preoperative image diagnosis in patients with esophageal cancer. PTACs with tracheal communication should be carefully left intact to avoid tracheal injury, while removal of BCs is recommended as a diagnostic and therapeutic measure.

## Limitations

This study had a small number of cases. It was not a clinical trial.

## Sources of funding

This work was partly supported by a Grant-in-Aid for Scientific Research (C) MEXT KAKENHI Grant Number 21K08729.

## Ethical approval

This case report was approved by the Ethics Committee of Toyama University Hospital.

## Consent

All patients fully understood condition of their disease and the surgical procedure they were agreeing to undergo, and a written informed consent was obtained from the patients for surgery. Written informed consent was obtained from the patient for publication of this case report and accompanying images. A copy of the written consent is available for review by the Editor-in-Chief of this journal on request.

## Research registration

Not applied.

## Guarantor

The Guarantor of this case report is T. Okumura.

## Provenance and peer review

Not commissioned, externally peer-reviewed.

## CRediT authorship contribution statement

T. Okumura produced the case report conception and design, obtained informed consent, and wrote the main manuscript body.

T. Okumura, T. Watanabe, K. Shibuya, T. Akashi, S. Sawada, K. Tazawa, and F. Yamagishi performed surgery.

K. Nomoto performed pathological diagnosis.

T. Watanabe, T. Miwa, T. Akashi, N. Kimura, N. Takeda, T. Uotani, H. Baba, K. Hirano, K. Shibuya, I. Hashimoto, S. Hojo, K. Matsui, I. Yoshioka, S. Sawada, K. Tazawa, F. Yamagishi, and T. Fujii participated in the patients' care and critically reviewed the manuscript.

## Declaration of competing interest

None of the authors have financial competing interests to disclose.
